# Automated analysis of limited echocardiograms: Feasibility and relationship to outcomes in COVID-19

**DOI:** 10.3389/fcvm.2022.937068

**Published:** 2022-07-22

**Authors:** Patricia A. Pellikka, Jordan B. Strom, Gabriel M. Pajares-Hurtado, Martin G. Keane, Benjamin Khazan, Salima Qamruddin, Austin Tutor, Fahad Gul, Eric Peterson, Ritu Thamman, Shivani Watson, Deepa Mandale, Christopher G. Scott, Tasneem Naqvi, Gary M. Woodward, William Hawkes

**Affiliations:** ^1^Department of Cardiovascular Medicine, Mayo Clinic, Rochester, MN, United States; ^2^Richard A. and Susan F. Smith Center for Outcomes Research in Cardiology, Beth Israel Deaconess Medical Center, Boston, MA, United States; ^3^Temple Heart and Vascular Center, Philadelphia, PA, United States; ^4^Ochsner Health System, New Orleans, LA, United States; ^5^Einstein Medical Center, Philadelphia, PA, United States; ^6^Department of Medicine, University of Pittsburgh, Pittsburgh, PA, United States; ^7^Department of Cardiovascular Medicine, Mayo Clinic, Scottsdale, AZ, United States; ^8^Department of Health Sciences Research, Mayo Clinic, Rochester, MN, United States; ^9^Ultromics Ltd., Oxford, United Kingdom

**Keywords:** echocardiography, artificial intelligence, deformation imaging, strain rate imaging, machine learning, COVID-19

## Abstract

**Background:**

As automated echocardiographic analysis is increasingly utilized, continued evaluation within hospital settings is important to further understand its potential value. The importance of cardiac involvement in patients hospitalized with COVID-19 provides an opportunity to evaluate the feasibility and clinical relevance of automated analysis applied to limited echocardiograms.

**Methods:**

In this multisite US cohort, the feasibility of automated AI analysis was evaluated on 558 limited echocardiograms in patients hospitalized with COVID-19. Reliability of automated assessment of left ventricular (LV) volumes, ejection fraction (EF), and LV longitudinal strain (LS) was assessed against clinically obtained measures and echocardiographic findings. Automated measures were evaluated against patient outcomes using ROC analysis, survival modeling, and logistic regression for the outcomes of 30-day mortality and in-hospital sequelae.

**Results:**

Feasibility of automated analysis for both LVEF and LS was 87.5% (488/558 patients). AI analysis was performed with biplane method in 300 (61.5%) and single plane apical 4- or 2-chamber analysis in 136 (27.9%) and 52 (10.7%) studies, respectively. Clinical LVEF was assessed using visual estimation in 192 (39.3%), biplane in 163 (33.4%), and single plane or linear methods in 104 (21.2%) of the 488 studies; 29 (5.9%) studies did not have clinically reported LVEF. LV LS was clinically reported in 80 (16.4%). Consistency between automated and clinical values demonstrated Pearson's R, root mean square error (RMSE) and intraclass correlation coefficient (ICC) of 0.61, 11.3% and 0.72, respectively, for LVEF; 0.73, 3.9% and 0.74, respectively for LS; 0.76, 24.4ml and 0.87, respectively, for end-diastolic volume; and 0.82, 12.8 ml, and 0.91, respectively, for end-systolic volume. Abnormal automated measures of LVEF and LS were associated with LV wall motion abnormalities, left atrial enlargement, and right ventricular dysfunction. Automated analysis was associated with outcomes, including survival.

**Conclusion:**

Automated analysis was highly feasible on limited echocardiograms using abbreviated protocols, consistent with equivalent clinically obtained metrics, and associated with echocardiographic abnormalities and patient outcomes.

## Introduction

The use of artificial intelligence (AI) as a method for automating medical image analysis has the potential to transform patient care (1). In echocardiography, AI applications have demonstrated significant value at numerous stages of the analysis pipeline, including automatic view classification (2–4), quantitative assessment of image quality (5, 6), automated contouring (7–9), assessment of regional wall motion (10), and disease classification (11–13). Notwithstanding the advantages of automated, high-throughput analysis, the benefits of AI driven analysis include savings of time (9, 14), improved prognostication (11, 15), reduced variability (16), and greater precision (6, 13). While the value of automated analysis is increasingly reported, validation of commercially available software with automated capabilities alongside clinical assessment remains limited (9, 17, 18). As a result, understanding of the capabilities and limitations of automated echocardiographic analysis remains incomplete.

Continued assessment of automated analysis using real-world data is essential to evaluate potential feasibility and relevance to clinical practice. In cases of severe infection, coronavirus disease 2019 (COVID-19) patients frequently present with prognostically significant cardiac involvement (19–21). Echocardiographic indices of both left- (LV) and right-ventricular (RV) function have been reported to effectively identify COVID-19 patients requiring urgent treatment or intervention (22), predict prognosis (18, 23) and allow longitudinal assessment (24). However, the use of limited echocardiographic acquisition protocols during the early stages of the pandemic (18, 21, 25, 26) often omitted some of the pre-requisites for advanced strain analysis [e.g., electrocardiogram monitoring and sufficient image quality from the three apical views (27)], limiting the information available to clinicians for patient risk stratification. Automated AI algorithms are capable of disease prediction (12, 13, 28) and functional quantification (6, 29), using limited or single-view images, without the requirement for additional work or expertise. However, the efficacy of automated analysis in patient assessment and risk stratification, including the potential impact of implementing automated analysis alongside routine practice, remains incompletely understood.

In this multi-site, retrospective study, we sought to evaluate (1) the feasibility of automated quantification of LV systolic function using limited echocardiograms from COVID-19 patients; (2) the agreement between automated quantification and clinical findings; (3) the association of automated assessment of the LV with in-hospital patient outcomes.

## Methods

### Patient population

This study was approved by the Institutional Review Boards and conducted among consecutive inpatient adults diagnosed with COVID-19 (positive antigen or polymerase chain reaction test) who underwent clinically indicated transthoracic echocardiography at six institutions: Beth Israel Deaconess Medical Center, Harvard Medical School (Boston); Temple University Hospital (Philadelphia); Einstein Medical Center (Philadelphia); Ochsner Medical Center (New Orleans); The University of Pittsburgh Medical Center; and Mayo Clinic Health System sites across Minnesota, Wisconsin, Florida, and Arizona, between February and December 2020. Only the first transthoracic echocardiogram performed during the hospital admission for COVID-19 was considered. Echocardiographic studies were included in the analysis if either an apical 4-chamber or apical 2-chamber image clip was available for analysis. Those with insufficient image quality to assess LV ejection fraction (EF) clinically and to evaluate the AI derived contours of the LV were excluded.

### Data collection

Patient baseline characteristics, medical history, and in-hospital outcomes were obtained at each site from review of electronic health records. These included patient demographics, presenting signs/symptoms, comorbidities at the time of initial hospital presentation, in-hospital sequelae, and echocardiographic findings. Outcomes included 30-day all-cause mortality, incident acute coronary syndrome (ACS), congestive heart failure (CHF), acute kidney injury, and major adverse cardiovascular and cerebrovascular events (MACCE), defined as the composite of ACS, CHF, stroke, coagulation disorder (disseminated intravascular coagulation or other acquired bleeding disorder), myocarditis, or pericarditis. Coronary artery disease (CAD) was defined as prior myocardial infarction (MI), coronary revascularization, or angiography showing stenosis >50% diameter. Echocardiographic variables were obtained from echocardiography reports and included (where available) qualitative assessment of cardiac function (regional wall motion abnormalities, LV size, LV wall thickness, left atrial size, RV size, LVEF, end-diastolic and end-systolic volumes, and LV longitudinal strain (LS).

### Echocardiographic analysis

As echocardiographic data was collected predominantly during the first wave of the pandemic, abbreviated and focused protocols were frequently utilized to minimize scan times and staff exposure risk (30), sometimes without placement of electrocardiographic leads (31, 32). Only studies that included at least one cardiac cycle from any of the apical views were considered. Echocardiographic examinations were performed using GE (Vivid E95 = 49.1%, Vivid S70 = 27.9%, Vivid E9 = 2.5%, Vivid IQ = 1.6%) and Philips (CX50 = 11.3%, EPIQ 7C = 3.9%, EPIQ CVx = 3.0%, iE33 = 0.7%) systems. Quantification of echocardiographic measures was obtained from two sources:

#### Clinically derived echocardiographic assessment

Quantitative assessment of LV function was obtained from clinical echocardiographic reports. The method of LVEF quantification (e.g., Simpson's biplane method of disks, single plane, linear, or visual estimation) was recorded. Protocols for echocardiographic acquisition and quantification were conducted according to local procedures and clinical standards in place at the time of data collection.

#### AI derived assessment

Quantitative assessment of LV function was obtained from automated AI driven echocardiography analysis algorithms (EchoGo Core v1.3.2, Ultromics Ltd, Oxford). LV LS was calculated as the average of the end-systolic longitudinal strain from apical 4- and 2-chamber views. Where one view was unavailable, single view longitudinal strain values were calculated. LV volumes and LVEF were determined using the Simpson's biplane method of disks. Where biplane LVEF was not feasible with both apical 4- and 2-chamber views, a single plane LVEF was calculated when feasible. The AI algorithms process apical 4- and 2-chamber images to automatically select cardiac cycles, contour the endocardial border, and calculate volumes, ejection fraction and longitudinal strain (18). Data for algorithm training were collected from an international dataset of clinically indicated echocardiograms, containing a range of patient pathologies (including coronary artery disease, heart failure, COVID-19, myocardial infarction, and prior cardiovascular disease) and were strictly independent of the participants of the current study.

### Feasibility of assessment

The endocardial border of apical 4- and 2-chamber images were automatically contoured by EchoGo Core and were presented to operators for approval. All operators held professional qualifications in echocardiography (e.g., British Society of Echocardiography or American Society of Echocardiography). All studies were processed through EchoGo Core, irrespective of image quality. A study was considered feasible for AI analysis if operators approved either apical 4- or 2-chamber views. Studies where AI analysis was not feasible (e.g., no contours approved for analysis) were included in the feasibility evaluation but not in the final analysis.

### Statistical analysis

Continuous variables were expressed as means ± standard deviations (±SD) or medians and interquartile ranges (IQR). Continuous data were compared between groups based on automated LS and EF using the Student's *t*-test or the Wilcoxon rank sum test, as appropriate. Categorical data was presented as counts and percentages and compared using the χ^*2*^ test. Pairwise comparisons of continuous and categorical data were conducted using paired *t*-tests and McNemar's test, respectively. Agreement analysis was conducted using Bland Altman statistics, linear Deming regression root mean square errors (RMSE), Pearson's correlation coefficients, and intraclass correlation coefficients (ICC). For agreement of LVEF between AI and clinical values, the analysis was conducted using comparable methods (e.g., biplane vs. biplane). Discordance between automated and clinically assessed LVEF was defined by an inter-method difference of >10%, which has been reported as the minimum detectable difference between observers (33, 34). For LVEF and LS, univariate logistic regression was performed to evaluate the association of AI and clinical echocardiographic measures with in-hospital outcomes and 30-day mortality. Patient origin was included in logistic regression equations to adjust for site related differences. Results from logistic regression models are reported as odds ratios (OR) and 95% confidence intervals. To account for the time to mortality, Cox proportional hazards regression was implemented. Kaplan-Meier estimates were used to provide a description of 30-day patient survival, with censoring after death, discharge or 30 days. Differences between survival curves were tested using the log-rank test. For LVEF, patients were classified into hyperdynamic (>70%, normal (55 to 70%), borderline (45 to 55%) and abnormal (<45%) (27, 35, 36). For LS, patients were classified as supranormal (<–20%), normal (−18 to −20%), borderline (−16 to −18%) and abnormal (>–16%) (27, 35, 36). Hyperdynamic and supranormal categories were included due to reports of being moderately prevalent in COVID-19 (24) with potential clinical significance (37, 38). All analysis was conducted using Python v3.9.7 in Spyder v5.1.5 using a two-tailed *p*-value <0.05 to define significance.

## Results

### Feasibility of AI analysis

Of 558 patient echocardiograms with at least one apical cardiac cycle, automated analysis of both LVEF and LS was feasible in 488 (87.5%). AI feasibility was 93.7% for the Mayo Clinic, 80.8% for Beth Israel Deaconess Medical Center, 100% for the University of Pittsburgh, 91.5% for Ochsner Medical Center, 72% for Temple University Medical Center and 89.7% for Einstein Medical Center (Supplementary Table 1). Reasons for rejection in the 70 studies included inability to fully assess endocardial border delineation in 66 (94.3%), incorrect frame selection in 2 (2.9%) and software errors in 2 (2.9%). There were 14 studies with clinically reported LVEF (1 assessed using biplane methods and 13 using linear methods) where automated analysis was not feasible.

Of the 488 accepted studies, AI analysis was performed with biplane method in 300 (61.5%) and single plane apical 4- or 2-chamber analysis in 136 (27.9%) and 52 (10.7%) studies, respectively. Clinical assessment of LVEF was recorded in 459 (94.1%) of the 488 studies at the time of the echocardiogram. Clinical LVEF was assessed using visual estimation in 192 (39.3%), biplane methods in 163 (33.4%), and single plane or linear methods in 104 (21.2%). LV LS was clinically reported in 80 (16.4%) patients.

### Patient characteristics

Baseline patient characteristics, demographics, and clinically derived echocardiographic parameters are reported in Table 1. The mean age was 62.2 ± 15.5 years and 279 (57.2%) were male. Indications for echocardiography included assessment of LV function in 223 (45.7%), hypoxemia in 88 (18.0%), arrhythmia in 46 (9.4%), suspected acute coronary syndrome in 26 (5.3%), assessment of RV function in 23 (4.7%), hypotension in 19 (3.9%), chest pain in 13 (2.7%), and others in 50 (10.2%).

**Table 1:** Baseline patient characteristics.

**Patient baseline characteristics**	**Value**
Age (Years)	62.24 ± 15.52
Male, *n* (%)	279 (57.4%)
BSA (m^2^)	2.11 ± 0.27
BMI (Kg/m^2^)	30.6 ± 6.68
Obesity, *n* (%)	136 (27.9%)
Systolic Blood Pressure (mm Hg)	124 ± 21
Diastolic Blood Pressure (mm Hg)	71 ± 14
Non-Hispanic White, *n* (%)	238 (50.0%)
Black or African American, *n* (%)	136 (28.6%)
Native American or Alaska Native, *n* (%)	29 (6.1%)
Hispanic, *n* (%)	96 (19.9%)
Diabetes Mellitus, *n* (%)	197 (40.4%)
Hypertension, *n* (%)	283 (58.0%)
Coronary Artery Disease, *n* (%)	77 (15.8%)
Cancer, *n* (%)	48 (9.8%)
Mechanical Ventilation During TTE, *n* (%)	126 (25.8%)
Vasopressor or Inotrope Use During TTE, *n* (%)	112 (23.0%)

*BMI, body mass index; BSA, body surface area; TTE, transthoracic echocardiography*.

### Comparison of AI and clinically derived assessment

**Figure 1:**
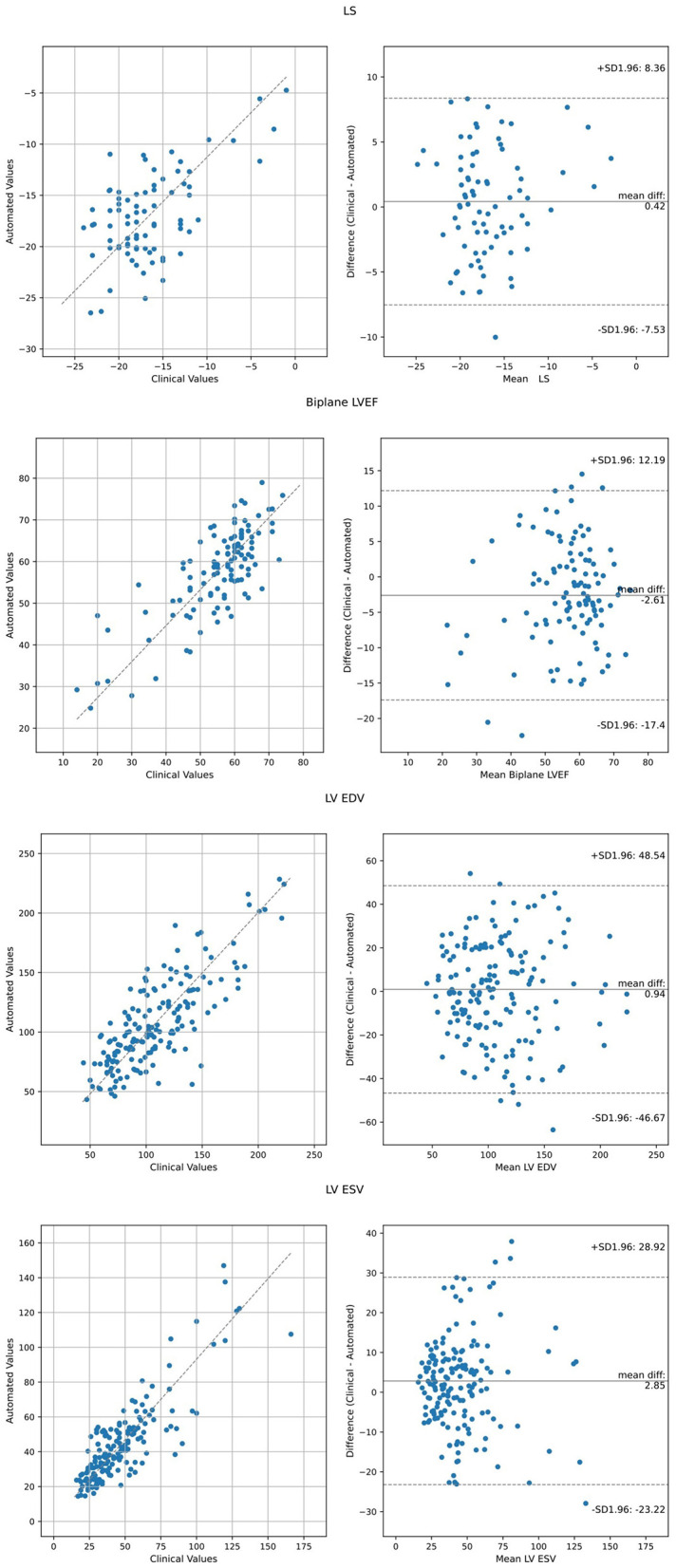
Agreement analysis between automated metrics of LV function relative to clinically derived values using Bland Altman analysis and Deming Regression. LVEF and LS values represent all available data, including biplane and single plane (either apical 4- or 2-chamber).

Comparison of the AI derived assessment to the values obtained from clinical echocardiography reports is reported in Table 2 and displayed in Figure 1. Agreement between automated and clinical LVEF using all available data had a mean difference of 0.91%, correlation coefficient 0.61, RMSE 11.3%, and an ICC 0.73. Inter-method agreement was highest when comparing like-for-like methods, with a correlation of 0.80 and an ICC of 0.85 for LVEF obtained using the biplane method. Agreement between automated and clinical LS using all available data (biplane or single plane for automated assessment) had a mean difference of −0.42%, correlation coefficient 0.73, RMSE 3.9% and an ICC 0.78. When restricting the comparison to cases where automated assessment was feasible on both apical 4- and 2-chamber views, agreement of LS demonstrated a mean difference −0.62%, correlation 0.73, RMSE 3.9%, and an ICC of 0.78.

**Table 2:** Agreement between automated metrics of LV function and values derived at the time of limited transthoracic echocardiogram.

	***n***	**Mean diff**	**LoA**	**Pearson's r**	**ICC**	**RMSE**
LS all	80	−0.417	7.945	0.725	0.782	3.876
Biplane LS	56	−0.62	7.738	0.739	0.791	3.877
LS Apical 4-chamber	75	−0.259	7.621	0.799	0.85	3.852
LVEF all	459	0.913	24.011	0.606	0.728	11.292
LVEF (biplane only)	112	2.606	14.792	0.796	0.848	7.024
LV EDV	168	−0.939	47.605	0.761	0.865	24.438
LV ESV	168	−2.85	26.068	0.82	0.897	12.78

*N comparisons indicates the number of datapoints available for extraction from clinical reports for comparison against the AI. LVEF all and LS all indicate data used from all comers, including all available methods of single view, biplane and triplane calculations. ICC, Intra-class correlation coefficient; LoA, Bland Altman limits of agreement; LS, longitudinal strain; LVEF, left ventricular ejection fraction; LV EDV, left ventricular end-diastolic volume; LV ESV, left ventricular end-systolic volume; RMSE, root mean square error*.

### Relationship of AI derived abnormalities with clinical echocardiographic abnormalities

The relationship of AI derived assessment and reported echocardiographic abnormalities was examined (Table 3). AI derived assessment identified 120 (24.6%) patients as having an LVEF <50% and 245 (50.2%) patients with LS >–16%. Patients with abnormal LVEF or LS determined by the AI method exhibited a significantly greater proportion of echocardiographic abnormalities in both the LV and RV, and more often had left atrial enlargement. The relationship of clinically assessed abnormality of LVEF and LS using the same cut points (<50% and >–16%, respectively) was similarly related to other reported echocardiographic abnormalities (Supplementary Table 2).

**Table 3:** Echocardiographic analysis of cardiac structure and function according to automated indices of LS (>–16%) and LVEF (<50%).

**Variable**	**N**	**All**	**LS** ≤ −**16%**	**LS**>**–16%**	***p*** **-value**	**LVEF** ≥**50%**	**LVEF**<**50%**	***p*** **-value**
Clinical LVEF (%)	459	56.35 ± 13.97	61.27 ± 8.92	51.52 ± 16.2	<0.001	60.12 ± 9.96	45.3 ± 17.75	<0.001
Clinical LVEF <50%	459	89 (19.3%)	14 (6.0%)	75 (32.0%)	<0.001	33 (10.0%)	56 (48.0%)	<0.001
Clinical LS (%)	80	−16.60 ± 4.66	−18.09 ± 3.09	−13.98 ± 5.75	<0.001	−17.71 ± 3.14	−11.38 ± 6.85	<0.001
Clinical LS >–16%	80	31 (38.8%)	13 (25.0%)	18 (62.0%)	<0.001	21 (32.0%)	10 (71.0%)	0.01
RWMSI	441	1.19 ± 0.43	1.06 ± 0.21	1.32 ± 0.54	<0.001	1.08 ± 0.23	1.54 ± 0.66	<0.001
RWMA	433	93 (21.5%)	24 (11.0%)	69 (33.0%)	<0.001	44 (13.0%)	49 (47.0%)	<0.001
Septal thickness (mm)	384	9.06 ± 4.62	8.97 ± 3.87	9.16 ± 5.31	0.69	9.1 ± 4.65	8.94 ± 4.54	0.78
Posterior wall thickness (mm)	382	8.94 ± 7.85	8.53 ± 3.62	9.37 ± 10.67	0.29	8.64 ± 3.96	9.92 ± 14.77	0.18
LV size	458							
Normal		409 (89.3%)	219 (96.0%)	190 (83.0%)	<0.001	327 (95.0%)	82 (73.0%)	<0.001
Enlarged		49 (10.7%)	10 (4.0%)	39 (17.0%)	<0.001	19 (5.0%)	30 (27.0%)	<0.001
LV hypertrophy	465	100 (21.5%)	32 (14.0%)	68 (29.0%)	<0.001	60 (17.0%)	40 (34.0%)	<0.001
Left atrial size	350							
Normal		268 (76.6%)	141 (81.0%)	127 (72.0%)	0.07	211 (81.0%)	57 (65.0%)	<0.001
Enlarged		82 (23.4%)	33 (19.0%)	49 (28.0%)	0.07	51 (19.0%)	31 (35.0%)	<0.001
Right ventricular function	448							
Normal		369 (82.4%)	204 (91.0%)	165 (74.0%)	<0.001	297 (88.0%)	72 (65.0%)	<0.001
Reduced		79 (17.6%)	21 (9.0%)	58 (26.0%)	<0.001	41 (12.0%)	38 (35.0%)	<0.001

*LS, longitudinal strain; LVEF, left ventricular ejection fraction; RWMSI, regional wall motion score index; RWMA, regional wall motion abnormality; LV, left ventricular*.

**Figure 2:**
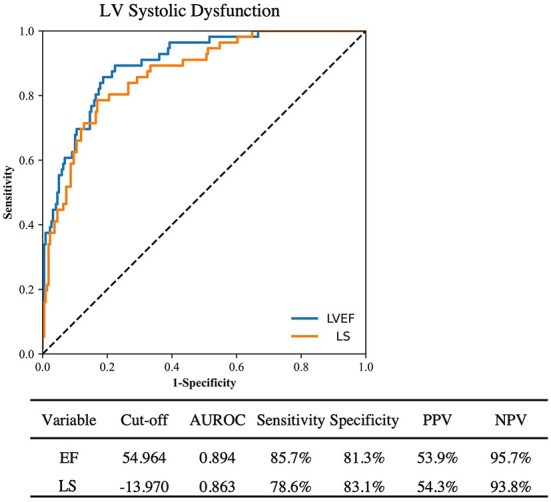
ROC curve analysis for detection of clinically reported LV systolic dysfunction by automated LVEF and LS. PPV, Positive predictive value; NPV, Negative predictive value.

ROC analysis of the ability of AI LVEF and LS to identify clinical systolic dysfunction (defined as a clinical LVEF <50%) achieved an area under the curve of 0.894 and 0.863, a sensitivity of 85.7% and 81.3%, and a specificity of 78.6% and 83.1%, respectively (Figure 2). Pairwise comparison between automated and clinical methods did not demonstrate any significant differences for LVEF (57 ± 12 vs. 56 ± 14%, *p* = 0.11) or LS (–17.0 ± 4.3 vs.−16.6 ± 4.7%, *p* = 0.36). Automated and clinical assessment of LS and LVEF demonstrated inter-method agreement in 70.0 and 79.6% of cases, respectively, when identifying LVEF <50% or LS >–16%, respectively. For LVEF, there were 61 (13.3%) cases where the automated assessment was <50% but the clinical values were >50% and 33 (7.2%) cases where the automated assessment was >50% and the clinical assessment was <50%. AI reported LVEF was different by a margin of more than 10% in 164 patients (36%) with 73% of these occurring in patients where the clinical assessment was performed using linear or manual methods. For LS, there were 11 (13.8%) of 80 cases where the automated assessment was >–16% and the clinical assessment was < –16% and 13 (16.3%) cases where the automated assessment was <–16% but the clinical assessment was >–16%. When comparing patients identified by clinically derived LVEF and LS, automated assessment characterized a significantly smaller proportion as abnormal [AI vs. clinical: LVEF: 56 (12.2%) patients vs. 89 (19.3%) patients, *p* < 0.001, LS: 18 (22.5%) patients vs. 31 (38.8%) patients, *p* < 0.001, Table 3].

### AI derived LVEF and LS and outcomes

During the first 30 days of hospitalization, death occurred in 103 (21.3%), acute coronary syndrome in 39 (8%), congestive heart failure in 49 (10%), and MACCE in 117 (24.0%). Using logistic regression adjusted by site (Table 4), automated LVEF and LS were associated with in-hospital death (LVEF *p* = 0.025, LS *p* = 0.03), ACS (LVEF *p* < 0.001, LS *p* < 0.001), CHF (LVEF *p* < 0.001, LS *p* < 0.001), acute kidney injury (LVEF *p* = 0.012, LS *p* = 0.03), and MACCE during hospital admission (LVEF *p* < 0.001, LS *p* < 0.001). Automated LVEF and LS were associated with mortality using Cox regression to account for increased risk over longer durations of hospitalization (LVEF *p* = 0.017, LS *p* = 0.033, Supplementary Table 3). Clinically derived LVEF and LS were associated with risk of CHF (LVEF *p* < 0.001, LS *p* = 0.001) and MACCE, (LVEF *p* < 0.001, LS *p* = 0.001) but not in-hospital death (LVEF *p* = 0.286, LS *p* = 0.158) or acute kidney injury (LVEF *p* < 0.43, LS *p* = 0.694). Clinical LVEF (*p* < 0.001) but not LS (*p* =0.118) was associated with ACS. Cox regression of clinical LVEF and LS was associated with mortality for LVEF (*p* = 0.019) but not for LS (*p* = 0.181, Supplementary Table 3).

**Table 4:** Site adjusted univariate logistic regression of automated and clinical LVEF and LS and clinical outcomes.

**Variable** + **Site**	**Odds Ratio**	**95% CI LL**	**95% CI UL**	**p-value**
**Death**				
Clinical LVEF	0.989	0.974	1.005	0.179
Automated LVEF	0.98	0.963	0.998	0.026
Clinical LS	1.094	0.965	1.241	0.161
Automated LS	1.051	1.003	1.1	0.035
**ACS**				
Clinical LVEF	0.954	0.935	0.973	<0.001
Automated LVEF	0.943	0.92	0.967	<0.001
Clinical LS	1.249	0.951	1.639	0.109
Automated LS	1.19	1.103	1.283	<0.001
**Congestive heart failure**				
Clinical LVEF	0.949	0.931	0.966	<0.001
Automated LVEF	0.94	0.919	0.962	<0.001
Clinical LS	1.625	1.153	2.289	0.005
Automated LS	1.16	1.085	1.24	<0.001
**MACCE**				
Clinical LVEF	0.956	0.941	0.97	<0.001
Automated LVEF	0.949	0.932	0.966	<0.001
Clinical LS	1.289	1.112	1.493	0.001
Automated LS	1.18	1.122	1.24	<0.001
**Acute kidney injury**				
Clinical LVEF	0.992	0.978	1.006	0.24
Automated LVEF	0.98	0.965	0.995	0.011
Clinical LS	1.02	0.921	1.13	0.704
Automated LS	1.044	1.003	1.087	0.034

*Clinical LVEF was assessed in 459, automated LVEF in 488, clinical LS in 80, and automated LS in 488. ACS: acute coronary syndrome, CI LL: 95% confidence interval lower limit, CI UL: 95% confidence interval upper limit, MACCE, major adverse cardiovascular and cerebrovascular events. LVEF, left ventricular ejection fraction; LS, longitudinal strain*.

**Figure 3:**
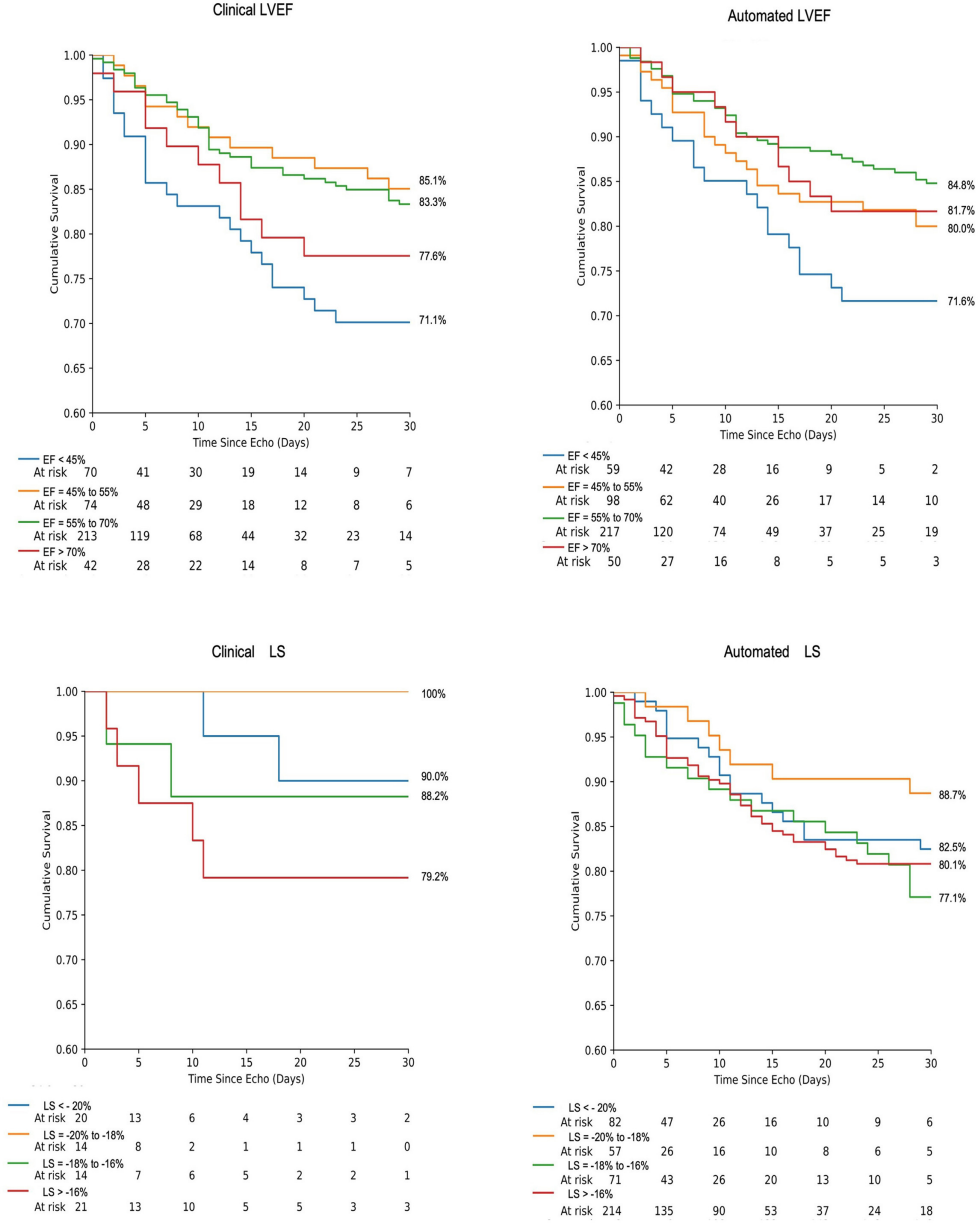
Kaplan-Meier survival analysis of 30-day mortality using LVEF and LS for both automated and clinically derived values according to strata of systolic dysfunction. Events are right-censored at 30 days.

When categorized into hyperdynamic, normal, borderline, and abnormal classes based on LVEF, survival rates were 77.6, 83.3, 85.1, and 71.1%, respectively, for clinical LVEF and 81.7, 84.8, 80.0, and 71.6%, respectively, for automated LVEF (Figure 3). When categorized into supranormal, normal, borderline, and abnormal classes based on LS, survival rates were 90.0, 100, 88.2, and 79.2%, respectively, for clinical LVEF and 82.5, 88.7, 77.1, and 80.1%, respectively, for automated LS. There were no significant differences in the overall survival between clinical and automated assessment at any level (supranormal/ hyperdynamic, normal, borderline, and abnormal *p* > 0.05). Kaplan Meier analysis demonstrated increased risk of death for those with abnormal LVEF, relative to normal LVEF, for both clinical (log-rank *p* = 0.004) and automated (log-rank *p* = 0.01) assessment. Relative to borderline LVEF, patients with abnormal LVEF demonstrated increased likelihood of death for clinical assessment (log-rank *p* = 0.01) but not for automated assessment (log-rank *p* = 0.19).

## Discussion

This study has evaluated the feasibility and clinical relevance of automated echocardiographic analysis software on a multi-site COVID-19 cohort, with an ethnically diverse population (50% non-Hispanic white), using limited echocardiographic studies. The main findings are: (1) Automated analysis of limited echocardiograms was feasible in 87.5% of patients in which at least one apical cardiac cycle was obtained, even under abbreviated protocols, with biplane analysis of LVEF and LS possible in 61.5% of patients; (2) Automated LVEF, LS, and volumes had good to excellent agreement with clinically derived values; (3) Automated LVEF and LS were able to stratify individuals with cardiac dysfunction, including clinically reported echocardiographic abnormalities; (4) Automated LVEF and LS were associated with adverse in-hospital and 30-day outcomes and were comparable to clinically derived assessment. These findings suggest that automated assessment of LV function is highly feasible under abbreviated protocols and provides prognostically relevant information while increasing the data available for risk stratification.

During the COVID-19 pandemic, it was quickly reported that cardiac complications were common in cases of serious infection and were associated with poor patient outcomes (19, 38–41). While the implementation of abbreviated protocols (31, 32, 42) enabled the identification of serious cardiac abnormalities relevant to patient care, the focus on brevity and minimized contact may have led to omission of important information relevant for risk stratification or quantitative assessment of cardiac function. In the present study, clinical assessment of LV function using biplane LVEF methods was performed in 33.4% and strain analysis was performed in 16.4% patients. By contrast, for AI assessment, biplane methods were used for both LVEF and LS in 61.5% of patients with single planar measures in all others. These findings do not necessarily reflect differences in the feasibility of biplane methods, but rather reflect the increased availability of information under the same circumstance, using the automated approach. Thus, automated analysis can facilitate streamlined image acquisitions (9, 17, 43) while increasing the reporting of strain analysis to compliment clinical decision making, without requiring additional bedside expertise or time for analysis. Additionally, the strength of the agreement between automated and clinical assessment increased when consistent quantitative methods were used. The consistency of quantification afforded by automated analysis could help to reduce the inherent variability associated with more manual approaches. With feasibility of 85% and good to excellent agreement between automated values and those obtained clinically, results from the present study provide additional evidence of the potential complementary nature of automated analysis, even in the setting of limited imaging protocols.

In addition to high feasibility, the ability to provide clinically relevant information to assist in patient risk stratification is a core requirement for medical devices. In the present study, patients with an automated LS of >–16% or LVEF of <50% were more likely to present with clinically identified cardiac abnormalities such as LV hypertrophy, regional wall motion abnormalities, and left atrial enlargement. Furthermore, both LS and LVEF were significantly associated with poor patient outcomes, with LS identifying patients with a 29% increased risk of MACCE (ACS, coagulation disorder, myocarditis, congestive heart failure, pericarditis, or stroke) per 1% increase (less negative) in LS. The risk of adverse in-hospital outcomes when stratified by LVEF or LS was largely consistent between clinical and automated assessments, providing further evidence of the validity of automated analysis. These findings are in line with recent work reporting that abnormal systolic LV function, as defined by AI derived LVEF and/or LS, are associated with adverse cardiac events and death (9, 17, 18). Importantly, automated and clinically derived assessment were comparable in their association with patient outcomes, suggesting that the prognostic value of this information is preserved despite automation (9). Together, these results indicate that automated analysis of LVEF and LS provides clinically relevant estimations and may provide additional information relevant to patient care.

Technological advances in both computing and instrumentation have steadily increased the availability of quantitative echocardiographic assessment in routine practice. Such assessment, including LS, have consistently demonstrated value for patient risk stratification and prognostication in a range of conditions (44–47). However, inter- and intra-operator variability present a challenge to clinical interpretation of quantitative data (34, 48). With the potential for substantial reductions of variability in analysis, AI provides a potential solution, but evidence of its feasibility remains limited. The feasibility of automated contours in comprehensive transthoracic echocardiographic datasets is reported to range from 60.6% (9) to 95% (17) which may, in part, be dependent upon image quality (6). Feasibility in the present study, which required at least one apical cardiac cycle, demonstrates the potential of automated algorithms even under challenging conditions with abbreviated protocols. However, further work, including prospective studies, is required to understand the feasibility and utility of automated analysis in different patient conditions undergoing limited echocardiograms, where image quality and availability are variable.

An important finding of this study was the discordance between automated and clinical characterization of abnormal systolic function by LVEF and LS. Automated and clinical assessment were discordant in 20 and 30% of cases for LVEF and LS, respectively. When comparing those deemed to have abnormal LVEF and LS using clinical assessment as the reference, classifications using automated assessment identified a significantly smaller population as abnormal (AI vs. clinical: LVEF: 12.2 vs. 19.3%, LS: 22.5 vs. 38.8%). However, this investigation did not demonstrate significant differences in relationship to overall mortality when stratified using clinical or automated assessment; thus, the clinical implications of such discrepancies are unclear. Such differences may reflect a bias from clinical interpretation of echocardiograms, where additional information can support differential diagnosis while the use of only apical views for automated assessment may underestimate patient risk. Indeed, the use of limited methods in the clinical estimation of LVEF (linear and visual estimation) contributed to 73% of the observed differences of more than 10% between AI and the clinical interpretation. Although no differences were observed in patient outcomes between AI and clinical methods, limited sample size and a relatively low event rate precluded further investigation of the clinical implications of discordance between the AI and the clinical interpretation. Further work is required to understand the relationship between automated assessment and clinical interpretation for risk stratification.

This study has several limitations. The retrospective nature of the investigation limits the generalizability of the findings to routine practice and further work is required to understand the real-time implications of the AI technology in varied populations. In addition, the clinical assessment of LVEF or LS was limited and as such, a comparison of AI feasibility to clinical feasibility was not possible. The limited sample of clinical LS values is a significant limitation when evaluating the association with patient outcomes. Automated assessment of RV function was not conducted and may have provided further prognostic information (18, 37). Secondly, the data consist of limited echocardiographic examinations conducted during the early COVID-19 pandemic; abbreviated examinations may have been of poorer quality due to risks of exposure. Echocardiographic protocols and clinical management of patients were conducted according to the local procedures in place at the time of data collection and regional and/or institutional bias may exist within the dataset. However, the resulting dataset is reflective of inter-site differences in practice and is a robust test of the AI algorithms. Finally, the cut-offs used for establishing the boundaries of normal, borderline and abnormal LVEF and GLS have varied between reports within the literature (27, 35, 36) and as such, the cut-offs utilized within the current study may not accurately distinguish normality from abnormality. Nevertheless, the implemented LVEF and GLS cut-offs provide further understanding of the findings from logistic regression models, whereby variables were modeled in a continuous manner.

## Conclusions

In a multi-center study conducted during a pandemic, automated analysis of ultrasound images was highly feasible, correlated with clinical observation, and associated with outcome.

## Data availability statement

The anonymized and aggregate raw data supporting the conclusions of this article will be made available by the authors, without undue reservation.

## Ethics statement

The studies involving human participants were reviewed and approved by Mayo Clinic Institutional Review Board. Written informed consent for participation was not required for this study in accordance with the national legislation and the institutional requirements.

## Author contributions

PP, WH, and GW designed the study. PP and WH drafted the manuscript. CS and WH organized the database and performed statistical analysis. JS and GP-H obtained local approval and data collection for Beth Israel Deaconess Medical Center. MK and BK obtained local approval and data collection for Temple Heart and Vascular Center. SQ and AT obtained local approval and data collection for Ochsner Health System. FG and EP obtained local approval and data collection for Einstein Medical Center. RT and SW obtained local approval and data collection for University of Pittsburgh. DM and TN obtained local approval and data collection for Mayo Clinic Scottsdale Arizona. PP oversaw local approval and data collection for Mayo Clinic Rochester Minnesota. All authors contributed to manuscript revision and read and approved the submitted version.

## Conflict of interest

GW and WH are employed by Ultromics Ltd. The remaining authors declare that the research was conducted in the absence of any commercial or financial relationships that could be construed as a potential conflict of interest. This study received funding from Ultromics Ltd. The funder had the following involvement with the study: The AI measurements in this study were performed by Ultromics Ltd.

## Publisher's note

All claims expressed in this article are solely those of the authors and do not necessarily represent those of their affiliated organizations, or those of the publisher, the editors and the reviewers. Any product that may be evaluated in this article, or claim that may be made by its manufacturer, is not guaranteed or endorsed by the publisher.
