# Supplementary material for: Automated analysis of limited echocardiograms: Feasibility and relationship to outcomes in COVID-19
Source: Front Cardiovasc Med. 2022 Jul 22;9:937068. doi: 10.3389/fcvm.2022.937068 (PMC9353267; doi:10.3389/fcvm.2022.937068)
Supplement: Supplementary file 1 [file Table_1.docx]

Supplementary Material

# Supplementary Tables

Supplementary Table 1. AI feasibility by site.

| ﻿Site | Accept | Reject |
| --- | --- | --- |
| Mayo Clinic | 239 | 16 |
| Beth Israel Deaconess Medical Center | 80 | 19 |
| University of Pittsburgh | 13 | 0 |
| Ochsner Medical Center | 54 | 5 |
| Temple University Hospital | 67 | 26 |
| Einstein Medical Centre | 35 | 4 |

Supplementary Table 2. Echocardiographic analysis of cardiac structure and function according to clinical indices of LS (>-16%) and LVEF (<50%)

| **Variable** | **N** | **All** | **LS ≤ -16%** | **LS > -16%** | **p** | **N** | **All** | **LVEF ≥ 50%** | **LVEF < 50%** | **p** |
| --- | --- | --- | --- | --- | --- | --- | --- | --- | --- | --- |
| Auto LVEF (%) | 80 | 59.28±10.71 | 61.97±7.95 | 55.02±13.05 | <0.001 | 459 | 57.26±12.14 | 60.22±10.06 | 44.94±12.39 | <0.001 |
| Auto LVEF < 50% | 80 | 14 (17.5%) | 4 (8.0%) | 10 (32.0%) | 0.01 | 459 | 117 (25.4%) | 61 (16.0%) | 56 (63.0%) | <0.001 |
| Auto LS (%) | 80 | -17.02±4.29 | -18.44±3.46 | -14.76±4.56 | <0.001 | 459 | -15.94±4.84 | -16.92±4.47 | -11.82±4.14 | <0.001 |
| Auto LS > -16% | 80 | 29 (36.2%) | 11 (22.0%) | 18 (58.0%) | <0.001 | 459 | 232 (50.4%) | 157 (42.0%) | 75 (84.0%) | <0.001 |
| RWMSI | 74 | 1.10±0.35 | 1.01±0.04 | 1.26±0.54 | <0.001 | 429 | 1.19±0.43 | 1.02±0.08 | 2.01±0.46 | <0.001 |
| RWMA | 71 | 6 (8.5%) | 2 (4.0%) | 4 (16.0%) | 0.22 | 421 | 90 (21.4%) | 25 (7.0%) | 65 (94.0%) | <0.001 |
| Septal Thickness | 75 | 11.09±4.80 | 10.24±1.42 | 12.45±7.39 | 0.05 | 382 | 9.06±4.63 | 9.01±4.7 | 9.29±4.35 | 0.65 |
| Posterior Wall Thickness | 75 | 10.31±3.07 | 9.54±1.57 | 11.52±4.29 | 0.01 | 380 | 8.93±7.87 | 8.94±8.46 | 8.89±3.98 | 0.96 |
| LV Size | 79 |  |  |  |  |  |  |  |  |  |
| Normal |  | 73 (92.4%) | 47 (96.0%) | 26 (87.0%) | 0.29 | 444 | 398 (89.6%) | 350 (97.0%) | 48 (57.0%) | <0.001 |
| Enlarged |  | 6 (7.6%) | 2 (4.0%) | 4 (13.0%) | 0.29 | 444 | 46 (10.4%) | 10 (3.0%) | 36 (43.0%) | <0.001 |
| LV Hypertrophy | 80 | 13 (16.2%) | 5 (10.0%) | 8 (26.0%) | 0.13 | 452 | 99 (21.9%) | 75 (20.0%) | 24 (28.0%) | 0.18 |
| Left Atrial Size | 57 |  |  |  |  |  |  |  |  |  |
| Normal |  | 49 (86.0%) | 31 (86.0%) | 18 (86.0%) | 1 | 342 | 262 (76.6%) | 224 (82.0%) | 38 (56.0%) | <0.001 |
| Enlarged |  | 8 (14.0%) | 5 (14.0%) | 3 (14.0%) | 1 | 342 | 80 (23.4%) | 50 (18.0%) | 30 (44.0%) | <0.001 |
| Right Ventricular Function | 77 |  |  |  |  |  |  |  |  |  |
| Normal |  | 65 (84.4%) | 44 (90.0%) | 21 (75.0%) | 0.16 | 436 | 361 (82.8%) | 317 (90.0%) | 44 (54.0%) | <0.001 |
| Reduced |  | 12 (15.6%) | 5 (10.0%) | 7 (25.0%) | 0.16 | 436 | 75 (17.2%) | 37 (10.0%) | 38 (46.0%) | <0.001 |

LS, longitudinal strain; LVEF, left ventricular ejection fraction; RWMSI, regional wall motion score index; RWMA, regional wall motion abnormality; LV, left ventricle.

Supplementary Table 3. Univariate Cox proportional hazards regression of LVEF and LS derived from clinical and automated assessment. Regression models have been adjusted for site related differences.

| **Variable** | **Odds Ratio** | **95% CI LL** | **95% CI UL** | **p** |
| --- | --- | --- | --- | --- |
| Death+site |  |  |  |  |
| Clinical LVEF | 0.984 | 0.971 | 0.997 | 0.019 |
| AI LVEF | 0.982 | 0.968 | 0.997 | 0.017 |
| Clinical LS | 1.087 | 0.962 | 1.228 | 0.181 |
| AI LS | 1.045 | 1.004 | 1.088 | 0.033 |

CI LL: 95% confidence interval lower limit, CI UL: 95% confidence interval upper limit, LS, longitudinal strain; LVEF, left ventricular ejection fraction; LV, left ventricular
